# Inhibition of Histone Deacetylases Attenuates Morphine Tolerance and Restores MOR Expression in the DRG of BCP Rats

**DOI:** 10.3389/fphar.2018.00509

**Published:** 2018-05-15

**Authors:** Xiao-Tao He, Kai-Xiang Zhou, Wen-Jun Zhao, Chen Zhang, Jian-Ping Deng, Fa-Ming Chen, Ze-Xu Gu, Yun-Qing Li, Yu-Lin Dong

**Affiliations:** ^1^Department of Human Anatomy, Histology and Embryology, K.K. Leung Brain Research Centre, Preclinical School of Medicine, The Fourth Military Medical University, Xi’an, China; ^2^Department of Periodontology, School of Stomatology, The Fourth Military Medical University, Xi’an, China; ^3^Student Brigade, The Fourth Military Medical University, Xi’an, China; ^4^Department of Neurosurgery, Tangdu Hospital, The Fourth Military Medical University, Xi’an, China; ^5^State Key Laboratory of Military Stomatology, Department of Orthodontics, School of Stomatology, The Fourth Military Medical University, Xi’an, China; ^6^Joint Laboratory of Neuroscience at Hainan Medical University and The Fourth Military Medical University, Hainan Medical University, Haikou, China

**Keywords:** bone cancer pain, morphine tolerance, HDACs, SAHA, MOR

## Abstract

The easily developed morphine tolerance in bone cancer pain (BCP) significantly hindered its clinical use. Increasing evidence suggests that histone deacetylases (HDACs) regulate analgesic tolerance subsequent to continuous opioid exposure. However, whether HDACs contribute to morphine tolerance in the pathogenesis of BCP is still unknown. In the current study, we explored the possible engagement of HDACs in morphine tolerance during the pathogenesis of BCP. After intra-tibia tumor cell inoculation (TCI), we found that the increased expression of HDACs was negatively correlated with the decreased expression of MOR in the DRG following TCI. The paw withdrawal threshold (PWT) and percentage maximum possible effects (MPEs) decreased rapidly in TCI rats when morphine was used alone. In contrast, the concomitant use of SAHA and morphine significantly elevated the PWT and MPEs of TCI rats compared to morphine alone. Additionally, we found that SAHA administration significantly elevated MOR expression in the DRG of TCI rats with or without morphine treatment. Moreover, the TCI-induced increase in the co-expression of MOR and HDAC1 in neurons was significantly decreased after SAHA administration. These results suggest that HDACs are correlated with the downregulation of MOR in the DRG during the pathogenesis of BCP. Inhibition of HDACs using SAHA can be used to attenuate morphine tolerance in BCP.

## Introduction

As advances in cancer therapy have extended the life expectancy of cancer patients, improving the quality of patient life is critically important (Siegel et al., 2017). Many common cancers, such as those arising from breast, prostate, kidney, and lung avidly metastasize to multiple skeletons and induce severe bone pain (Mantyh, 2013; Vignani et al., 2016). Bone cancer pain (BCP) is one of the most difficult types of chronic pain to fully control and thus negatively affects the quality of life of patients (Weilbaecher et al., 2011; Falk and Dickenson, 2014). Opioids are the mainstay for analgesic therapy in advanced cancer pain according to a 3-step “analgesic ladder” promulgated by WHO in 1986. However, the repeated use of opioids can easily cause analgesic tolerance, the hallmark feature of which is opioid dose escalation to maintain adequate pain relief (King et al., 2005; Trang et al., 2015).

It has been reported that patients suffering from BCP require significantly higher doses of morphine than individuals with inflammatory pain. Animal experiments have shown that doses of morphine required to relieve BCP are 10 times higher than those required to block peak inflammatory pain (Luger et al., 2002; Mercadante and Fulfaro, 2007; Glauben et al., 2009; Yao et al., 2016). Opioid effects (e.g., analgesia and tolerance) are predominantly mediated by the μ-opioid receptor (MOR) (Yao et al., 2016). Several studies have shown that MOR expression is significantly decreased in the DRG in a mouse model of BCP, whereas MOR expression in the DRG of a mouse model of inflammatory pain was unchanged (Mao et al., 1995; Yamamoto et al., 2008; Yao et al., 2016). This finding suggests that patients with BCP prone to morphine tolerance may be due to the downregulation of MOR in the DRG. Histone deacetylases (HDACs) are emerging tools for the modulation of gene transcription (Sahbaie et al., 2016). Compelling evidence has suggested that HDACs are involved in MOR gene silencing (Hwang et al., 2007; Hou et al., 2017). Our previous research has indicated that the hypoacetylation state of histones H3 and H4 contributes to the decreased expression of MOR in the DRG during the development of BCP (Zhu C. et al., 2017). Thus, we hypothesize that the downregulation of MOR in the DRG of BCP may be correlated with the increased expression of HDACs, and inhibition of HDACs could enhance MOR expression to attenuate morphine tolerance.

Therefore, the objectives of the current study were to explore the potential interactions between HDACs and MOR in the DRG in the pathogenesis of BCP and to examine the efficacy of SAHA, a clinically used HDACi, on morphine tolerance during the pathogenesis of BCP. First, we examined the expression of HDACs and MOR in the DRG and their correlations during the pathogenesis of BCP. Then, we investigated the analgesic effects of morphine on BCP and ascertained whether co-administration of SAHA with morphine can attenuate morphine tolerance in BCP. Finally, we determined the effects of SAHA on MOR expression following tumor cell inoculation (TCI) with or without morphine treatment.

## Materials and Methods

### Animals

Female Sprague-Dawley (SD) rats (weight, 180–200 g) were provided by the Laboratory Animal Center of the Fourth Military Medical University (FMMU). All animal procedures were carried out in accordance with the recommendation of the Principles of Laboratory (NIH Publication no. 85-23, revised 1985) and the ethics committee of the International Association for the Study of Pain (Zimmermann, 1983). The study was approved by the ethics committee for Animal Care and Use Committees of the FMMU prior to the onset of the experiments (Permit number: 20160101). Two to three rats were housed per cage in pathogen-free conditions under controlled ambient temperature (22 ± 2°C) and photoperiods (12-h light-dark cycle). All rats were allowed to acclimate to these conditions for 5 days before beginning baseline testing. Animals were randomly divided into groups (*n* = 6–8). For each group of experiments, animals were age- and body weight-matched.

### Intrathecal Implantation

After baseline tests, intrathecal implantation was performed as described in previous study (Xu et al., 2014; Xu H. et al., 2015; Wang et al., 2016). Briefly, an intrathecal PE-10 catheter (Becton Dickinson, San Jose, CA, United States) was inserted into the subarachnoid space of the lumbar enlargement under complete anesthesia. To confirm the success of catheterization, 10 μL of lidocaine (2%) was injected through the catheter on the next day. Rats showing immediate hind limb paralysis after injection were considered to have successful placement and were prepared for the BCP model. At the end of each experiment, the position of the polyethylene tubing in the intrathecal space was visually verified by exposing the lumbar spinal cord. Data from rats with incorrect PE tubing position were discarded from the study.

### Cell Preparation

Walker 256 breast carcinoma cells were purchased from American Type Culture Collection (ATCC, United States). Briefly, Walker 256 cells (2 × 10^7^ in 0.5 mL) were injected into the abdominal cavity of female SD rats. After 7–10 days, the produced ascites (approximately 2 mL) was collected and centrifuged at 1,000 *g* for 3 min. The cells in the ascites were then washed with 10 mL of sterile phosphate-buffered saline (PBS) three times. The samples were subsequently suspended in sterile PBS to a final concentration of 5 × 10^5^ cells/10 μL. The cells were kept on ice before being used. The same final concentration of cells was boiled for 20 min and used as the Sham group.

### Bone Cancer Pain Model

The BCP model was performed at 7 days after intrathecal implantation. As described in our previous studies (Chen et al., 2013; Hu et al., 2016), rats were anesthetized with sodium pentobarbital (50 mg/kg, i.p.), and a minimal skin incision (8–10 mm) was then made to expose the tibia. 10 μL of the Walker 256 carcinoma cell suspension (5 × 10^5^ cells in sterile PBS solution) was slowly injected into the intramedullary cavity of the medial aspect of the tibia. The syringe was then removed, and the injection hole was plugged immediately with bone wax to prevent any escape of tumor cells. In the Sham group, the same procedures were followed, except that an equal volume of heat-killed carcinoma cells was injected instead of normal carcinoma cells.

### Drug Administration

Drug doses were selected based on previous reports and our preliminary experiments (Wang et al., 2016; Xu et al., 2014; Xu J. et al., 2015). When the PWT of TCI rats were significantly decreased on post-operative day (POD) 8, 20 μg/kg morphine diluted in 10 μL PBS (Northeast Pharmaceutical Group, Shenyang, China) was administered intrathecally (i.t., diluted with sterile PBS) twice daily (at 8:00 am and 6:00 pm) from POD 8 to POD 14. Sham rats treated with the same volume of vehicle solution (PBS) were used as the control group.

SAHA (purchased from Selleck) was dissolved in dimethylsulfoxide (DMSO, Sigma, St. Louis, MO, United States), diluted in physiological saline to a final concentration of 5 mg/mL (in 5% v/v DMSO) and stored at -20°C. SAHA (20 mg/kg, i.p.) was injected intraperitoneally daily 30 min after behavioral tests from POD 8 to POD 14. Sham rats treated with a vehicle solution containing 5% DMSO were used as a control for the SAHA group.

### Radiological Analysis

To confirm cancer development in the tibia, TCI and Sham rats were radiographed on POD 14. Rats were anesthetized with sodium pentobarbital (50 mg/kg, i.p.), and placed prone on an X-ray film (Henry Schein blue sensitive film, Henry Schein, Melville, NY, United States), and then exposed to an X-ray source (Emerald 125) for 1/20 s at 40 KVP. The X-ray film was developed in a film developer (Konica SRX-101A, Konica Minolta, Tokyo, Japan).

### Bone Histology

On POD 14, the ipsilateral tibias were removed after euthanasia and decalcified in 10% ethylene diamine tetraacetic acid (EDTA; Sigma) solution for 3–4 weeks. The bones were rinsed, dehydrated, embedded in paraffin, cut into 7-μm cross-sections using a rotary microtome (Reichert-Jung 820, Cambridge Instruments GmbH, Nussloch, Germany), and stained with hematoxylin and eosin (H&E) to visualize normal marrow elements and cancer cells under bright field microscopy.

### MicroCT Analysis

Tibias were carefully harvested, fixed in 4% paraformaldehyde and stored at -80°C until scanning with a high-resolution microCT (GE Healthcare, Madison, WI, United States). The scans were performed in the long axis of the diaphysis with the following basic scanning parameters: voltage of 80 kVp, current of 80 μA, exposure time of 3000 ms, total rotation angle of 360° and rotation angle increment of 0.4°. Analysis was performed using Micview V2.1.2 software. The 3D data sets were low-pass filtered and segmented with a fixed threshold filter (1000 mg HA/cm^3^) according to current guidelines (Bouxsein et al., 2010). For the quantitative analysis of bone destruction, the volume of interest (VOI) was defined as a round yellow region that started at a distance of 0.1 mm from the top end of the growth plate and extended to the proximal end of the tibia for a distance of 1.5 mm. Only spongiosa was included in the VOI. The bone mineral density (BMD) of the trabeculae was then calculated based on microCT scanning.

### Mechanical Allodynia Test

The animals were habituated to the testing environment for 5 days. Baseline testing was performed before and after intrathecal implantation. Animals were discarded in the present study when the difference of the baseline before and after intrathecal implantation was greater than 4 g. The average of these two baseline tests was recorded as the baseline data. Rats were placed on wire mesh platforms in clear cylindrical plastic enclosures 10 cm in diameter and 40 cm in height. After 30 min of acclimation, PWT were tested using Von Frey filaments (Stoelting, Kiel, WI, United States) by experimenters who were blinded to group assignment. Fibers of sequentially increasing stiffness (0.4, 0.6, 1.0, 2, 4, 6, 8, 10, 15, and 26 g) were applied to the plantar surface, pressed upward to cause a slight bend in the fiber and left in place for 5–6 s. Each filament was applied 10 times, and the minimal value that caused at least six responses was recorded as the paw withdrawal threshold (PWT). Acute withdrawal, biting, licking or shaking of the ipsilateral hind limb and vocalization were considered positive signs of withdrawal.

### Hot Plate Test

A hot plate test was carried out to assess the chronic morphine tolerance according to previous research (Cai et al., 2016; Hu et al., 2016). Rats were placed on a 55°C hot plate (Socrel hot-plate model DS37, Ugo Basile, Italy). The response latency to either a hind paw lick or a jump was recorded. In the absence of a response, a cutoff time of 20 s was set to avoid tissue damage. Before morphine administration, the hot plate latency was measured three times. The average was used as the pre-drug response latency. To confirm the analgesic effects of morphine, the hot plate latency was measured three times at 30 min after each injection. The average was then used as the post-drug response latency. The percentage of maximal possible effect (MPE) was calculated, where MPE (%) = ([post-drug maximum response latency-pre-drug response latency]/[cutoff time {20 s}- pre-drug response latency]) × 100%.

### Western Blot

Animals were anesthetized with pentobarbital (50 mg/kg, i.p.) and rapidly sacrificed by decapitation. The L4 and L5 dorsal root ganglia (DRG) were subsequently dissected on ice. Then, total protein was extracted using lysis buffer (Beyotime, Shanghai, China) with a mixture of proteinases (Roche, Tucson, AZ, United States). Protein concentrations of the lysates were estimated and equalized using the bicinchoninic acid (BCA) method (with reagents from Thermo Scientific, Rockford, IL, United States). The electrophoresis samples were then heated at 100°C for 5 min and loaded onto 10% SDS–polyacrylamide gels with standard Laemmli solutions (Bio-Rad Laboratories, Hercules, CA, United States). The separated proteins were subsequently electroblotted onto a polyvinylidene difluoride membrane (Millipore, Billerica, MA, United States). The membranes were placed in a blocking solution containing Tris-buffered saline with 0.02% Tween-20 (TBS-T) and 4% non-fat milk for 1 h and then incubated overnight under gentle agitation with primary antibodies. The following primary antibodies were used: rabbit anti-HDAC1 IgG (1:1000, Cell Signaling Technology, Beverly, MA, United States), rabbit anti-HDAC2 IgG (1:1000, Cell Signaling Technology), and rabbit anti-MOR IgG (1:1000, Abcam, Cambridge, MA, United Kingdom). The membranes were then developed using enhanced chemiluminescence reagents (Amersham Life Science, Amersham, United Kingdom) after incubation with horseradish peroxidase-conjugated anti-rabbit secondary antibody (1:5000; Amersham Pharmacia Biotech, Piscataway, NJ, United States). Data were analyzed using a Molecular Imager (ChemiDoc XRS; Bio-Rad) and the associated software ImageJ (National Institute of Health, Bethesda, MD, United States).

### Immunofluorescent Staining

On POD 14, rats were anesthetized and perfused through the ascending aorta with 0.05 M PBS, followed by 4% (w/v) paraformaldehyde in 0.1 M phosphate buffer (PB). The L4 and L5 DRGs were immediately removed and dehydrated for 24 h in 0.01 M PB containing 30% sucrose at 4°C. Transverse frozen DRG sections (15 μm in thickness) were cut with a cryostat (Leica CM1800; Heidelberg, Germany) and collected serially into several dishes. Each dish contained a complete set of serial sections that were processed for immunofluorescence staining. The sections in the dish were rinsed in 0.01 M PBS (pH 7.3) 3 times (10 min each) and blocked with 2% normal donkey serum in 0.01 M PBS containing 0.3% (v/v) Triton X-100, 0.02% (w/v) sodium azide, and 0.12% (w/v) carrageen (pH 7.4, all purchased form Sigma) for 1 h at room temperature (RT, 20–25°C). The sections were incubated overnight at 4°C with the following primary antibodies: rabbit anti-HDAC1 IgG (1:300, Sigma), rabbit anti-HDAC2 IgG (1:300, Sigma), and mouse anti-MOR IgG (1:200, Sigma). For double immunofluorescence, sections were incubated with a mixture of two primary antibodies, followed by a mixture of the two respective secondary antibodies: Alexa 488 donkey anti-rabbit IgG (1:500, Invitrogen, Carlsbad, CA, United States) and Alexa 594 donkey anti-mouse IgG (1:500; Invitrogen). Confocal images were obtained using a confocal laser microscope (FV1000; Olympus, Tokyo, Japan), and digital images were captured with a FluoView 1000 microscope (Olympus). The specificity of the staining was tested on sections in the second dish by omission of the primary specific antibodies. No immunoreactive products were found on these sections. To determine the number of neurons double-labeled for MOR and HDAC1 or HDAC2, only neurons with clearly visible nuclei were counted. Neurons were determined as MOR/HDAC1/HDAC2 positive if the signal intensity was three-fold higher than the background using Stereo Investigator software (MicroBrightField, Williston, VT, United States). The double-labeled neurons were then counted in at least five randomly selected DRG sections per animal. To prevent the duplicate counting of neuron cell bodies, sections that were 50 μm apart were counted for each DRG. The work was performed by experimenters who were blinded to group assignment.

### Statistical Analyses

All data were collected by researchers who were blinded to the surgeries and reagents used. GraphPad Prism version 5.01 for Windows (San Diego, CA, United States) was used to conduct all statistical analyses. Nociceptive behavioral tests completed over time among groups were tested with the non-parametric Friedman test for repeated measures followed by *q* test. Differences in Western blot and immunofluorescence values over time for each group were tested using the non-parametric Mann–Whitney *U* test. Pearson correlation was used for the linear correlation analysis. Statistical significance was considered when *p* < 0.05.

## Results

### Severe Bone Destruction and Decreased PWT Following TCI

**FIGURE 1:**
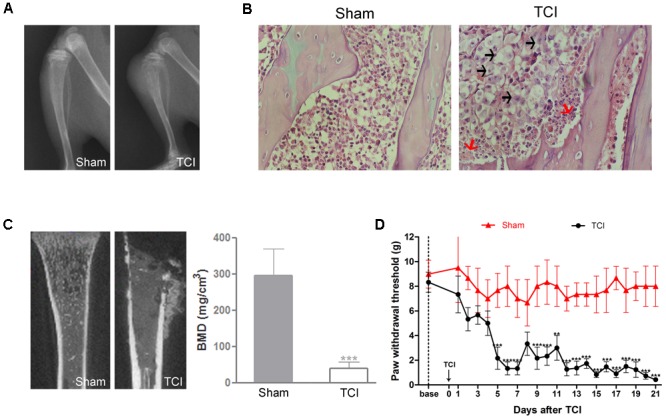
Tumor cell inoculation (TCI)-induced bone destruction and mechanical allodynia. **(A)** Radiographs of tibia bone in the Sham and the TCI rats on POD 14. **(B)** H&E staining of the trabecular bone in tibia marrow cavity of the Sham and the TCI group on POD 14. Black arrows indicate the infiltrated cancer cells; red arrows indicate osteoclastic resorption pits. Original magnification: 400× **(C)** Micro-CT scanning of proximal tibias in the groups of the Sham and the TCI on POD 14. Bone mineral density (BMD) of trabecular bone of the tibia in the TCI was significantly decreased compared to that in the Sham group (*n* = 4 rats in each group). **(D)** PWT of the TCI rats was significantly decreased following TCI (*n* = 6 rats in each group). Data are expressed as the mean ± SEM. ^∗∗^*P* < 0.01, ^∗∗∗^*p* < 0.001 versus the Sham group.

Radiology data showed a large area of radiolucent lesions in the proximal epiphysis of the tibias in the TCI group on POD 14, but no change was observed in the tibias in the Sham group (**Figure 1A**). The H&E staining showed that the tibial marrow cavity of TCI rats was filled with cancer cells (black arrows), and osteoclastic resorption pits (red arrows) on the surface of trabecular bone. In contrast, no cancer cells or osteoclasts were observed in the tibia marrow cavity of Sham rats (**Figure 1B**). Similarly, microCT scanning showed a large area of radiolucent lesions in the epiphysis and erosion of cortical bone in the tibia of TCI rats. The BMD of the tibia in the TCI group was decreased significantly compared to that in the Sham group (**Figure 1C**). Meanwhile, the PWT of ipsilateral hind paws was continually decreased on POD 5, and then remained at a significantly lower level until the experiment ended (**Figure 1D**).

### Decreased MOR Expression in the DRG Following TCI

**FIGURE 2:**
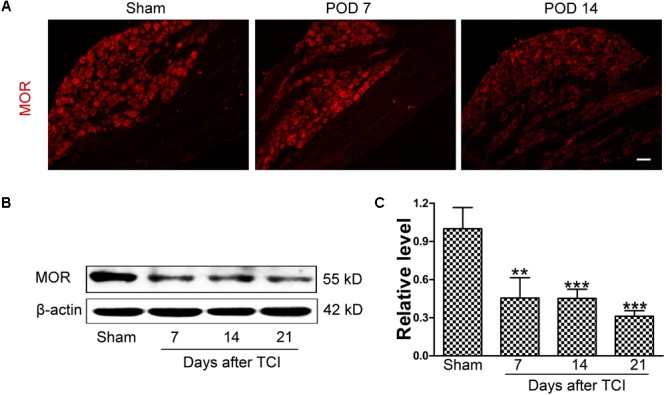
The downregulation of MOR in the DRG following TCI. **(A)** Immunofluorescent staining of MOR (red fluorescence) in the DRG at multiple time points (Sham, POD 7, and POD 14). Scale bar: 100 μm. Representative bands **(B)** and quantitative analysis of MOR expression **(C)** in the DRG at multiple time points (Sham, POD 7, POD 14, and POD 21) following TCI (*n* = 4 in each group). Analysis was based on mean gray values, normalized to β-actin. Data were expressed as mean ± SEM. ^∗∗^*p* < 0.01, ^∗∗∗^*p* < 0.001 versus the Sham group.

Immunofluorescent staining showed that the distribution of MOR-like immunoreactivities (red fluorescence) was observed in the DRG. The immunofluorescence intensity of MOR was continually decreased in the DRG at multiple time points (sham, POD 7 and POD 14) following TCI (**Figure 2A**). Consistently, Western blot analysis showed that the MOR expression in the DRG was decreased rapidly and significantly from POD 7 to POD 21 (**Figures 2B,C**).

### The Correlations of the Expression of MOR and HDACs in the DRG Following TCI

**FIGURE 3:**
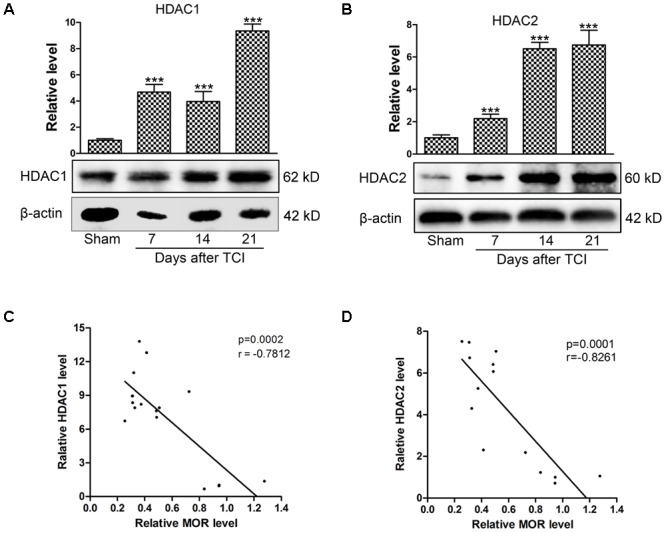
The negative correlation between the HDACs and MOR in the DRG following TCI. Representative bands analyzed by Western blot and quantitative analysis of expression levels of HDAC1 **(A)** and HDAC2 **(B)** at multiple time points (Sham, POD 7, POD 14, and POD 21) following TCI (*n* = 4 in each group). Analysis was based on mean gray values, normalized to β-actin. Data are expressed as the mean ± SEM. ^∗∗∗^*p* < 0.001 versus the Sham group. **(C,D)** The correlation analysis between MOR and HDAC1 (**C**, *r* = –0.7812, *p* < 0.001) or HDAC2 (**D**, *r* = –0.8261, *p* < 0.001) in the DRG of TCI rats.

To explore whether HDACs contribute to the decreased MOR expression in the DRG of TCI rats, the expression of HDAC1 and HDAC2 were analyzed by Western blot. Our data showed that the expression levels of HDAC1 and HDAC2 in the DRG were increased significantly from POD 7 to POD 21 compared with those of Sham rats. The upregulation of HDAC1 (**Figure 3A**) and HDAC2 (**Figure 3B**) persisted until POD 21. The relationship between the expression of HDACs and MOR was analyzed subsequently. Linear correlation analysis showed that both HDAC1 (**Figure 3C**, *r* = -0.7812, *p* < 0.001) and HDAC2 expression levels (**Figure 3D**, *r* = -0.8261, *p* < 0.001) were negatively correlated with MOR protein expression.

### The Analgesic Effects of Morphine on TCI-Induced Mechanical Allodynia

**FIGURE 4:**
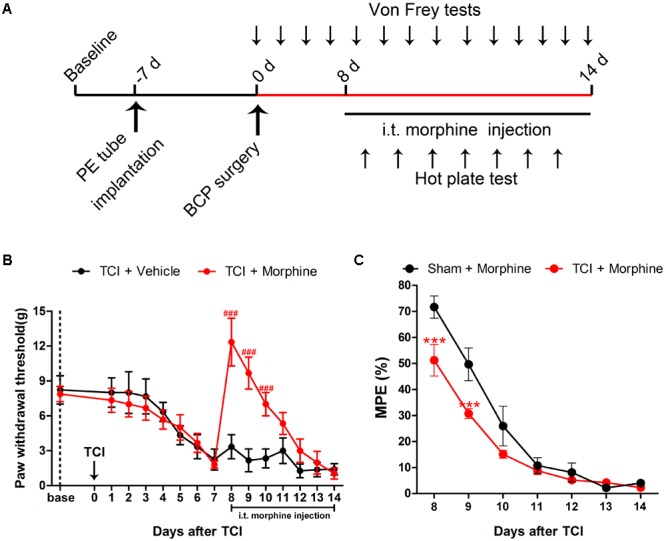
The analgesic effects of morphine (i.t., 20 μg/kg) on TCI-induced mechanical allodynia. **(A)** Experimental paradigms. **(B)** Comparisons of PWT of ipsilateral hind paws of rats in the TCI + Vehicle group and the TCI + Morphine group (*n* = 6 rats in each group). **(C)** Comparisons of MPE of rats in the Sham + Morphine group and the TCI + Morphine group (*n* = 6 rats in each group) following morphine administration from POD 8 to POD 14. Data are expressed as the mean ± SEM. ^###^*p* < 0.001 versus the TCI + Vehicle group and ^∗∗∗^*p* < 0.001 versus the Sham + Morphine group.

To assess the analgesic effects of morphine on BCP, 20 μg/kg morphine was injected i.t. twice daily from POD 8 to POD 14 (**Figure 4A**). Compared with the TCI + Vehicle group, i.t. administration of morphine significantly elevated the PWT of ipsilateral hind paws of TCI rats on POD 8. However, the PWT of TCI + Morphine was subsequently decreased on a gradual manner. No significant differences were observed between the PWT of the TCI + Vehicle group and the TCI + Morphine group from POD 11 to POD 14 (**Figure 4B**). Morphine tolerance in the Sham + Morphine and TCI + Morphine groups was measured by hot plate test following morphine administration from POD 8 to POD 14. The MPEs in both groups were decreased gradually (**Figure 4C**). However, the MPEs of the TCI + Morphine group were significantly lower than those of the Sham + Morphine group during the first 2 days of morphine exposure (**Figure 4C**).

### The Effects of SAHA Administration on Morphine Tolerance in BCP

**FIGURE 5:**
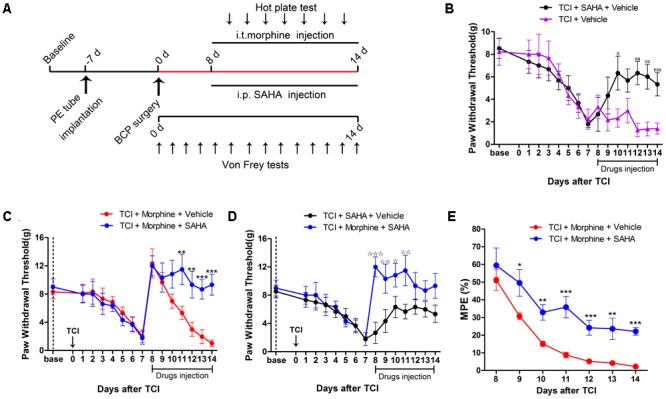
SAHA administration attenuated morphine tolerance in BCP. **(A)** Experimental paradigms. **(B)** Comparisons of PWT of rats in the TCI + SAHA + Vehicle and the TCI + Vehicle group (*n* = 6 rats in each group). **(C)** Comparisons of PWT of rats in the TCI + Morphine + Vehicle and the TCI + Morphine + SAHA group (*n* = 6 rats in each group). **(D)** Comparisons of PWT of rats in the TCI + SAHA + Vehicle and the TCI + Morphine + SAHA group (*n* = 6 rats in each group). **(E)** Comparisons of MPE of rats in the TCI + Morphine + Vehicle and the TCI + Morphine + SAHA (*n* = 6 rats in each group). Data are expressed as the mean ± SEM. ^#^*p* < 0.05, ^##^*p* < 0.01, ^###^*p* < 0.001 versus the TCI + Vehicle group, ^∗^*p* < 0.05, ^∗∗^*p* < 0.01, ^∗∗∗^*p* < 0.001 versus the TCI + morphine + Vehicle group, *p < 0.05, **p < 0.01, ***p < 0.001 versus the TCI + SAHA + Vehicle group.

Given that the decreased MOR expression was negatively correlated with the increased HDAC expression in the DRG following TCI, we then examined whether inhibition of HDACs by SAHA, a clinically used HDAC inhibitor, can attenuate morphine tolerance during the pathogenesis of BCP (**Figure 5A**). First, we examined the analgesic effects of SAHA alone on BCP. When 20 mg/kg SAHA was i.p. injected from POD 8 to POD 14, the PWT of the TCI + SAHA + Vehicle rats was significantly increased from POD 12 to POD 14 compared with the TCI + Vehicle group (**Figures 5A,B**). Then we explored the concomitant administration of morphine and SAHA on BCP. TCI rats were treated with both morphine (20 μg/kg, i.t., twice daily) and SAHA (20 mg/kg, i.p., once daily) from POD 8 to POD 14 (**Figure 5A**). Compared with the TCI + Morphine + Vehicle group, the PWT of the TCI + Morphine + SAHA rats was significantly increased from POD 11 to POD 14 (**Figure 5C**). In addition, the TCI + Morphine + SAHA group showed significantly higher PWT than the TCI + SAHA + Vehicle group from POD 8 to POD 11 (**Figure 5D**). Finally, we analyzed the effects of SAHA on morphine tolerance during the pathogenesis of BCP by hot plate test. The quantitative analysis showed that the MPEs of the TCI + Morphine + SAHA group were also significantly higher than that of the TCI + Morphine + Vehicle group from POD 9 to POD 14, which indicated that the SAHA administration could significantly attenuate the development of morphine tolerance in BCP (**Figure 5E**).

### The Effects of SAHA on HDAC and MOR Expression in the DRG of TCI Rats With or Without Morphine Treatment

**FIGURE 6:**
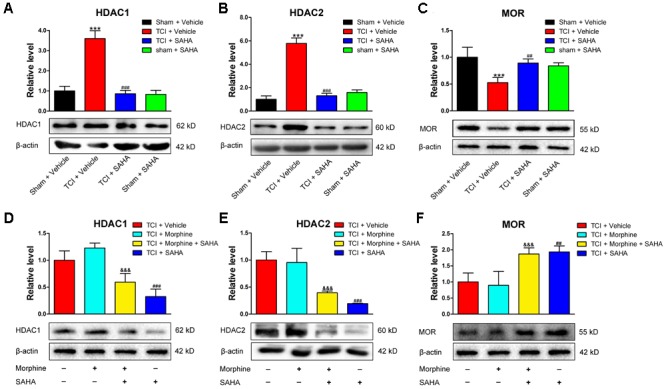
SAHA administration (i.p., 20 mg/kg) reversed HDACs and MOR expression in the DRG following TCI with or without morphine treatment. Quantitative analysis by Western blot and representative bands of expression levels of HDAC1 **(A)**, HDAC2 **(B)**, and MOR **(C)** in the DRG of the Sham + Vehicle, the TCI + Vehicle, the TCI + SAHA and the Sham + SAHA group (*n* = 4 in each group). Quantitative analysis by Western blot and representative bands of expression levels of HDAC1 **(D)**, HDAC2 **(E)**, and MOR **(F)** in the DRG of the TCI + Vehicle, the TCI + Morphine, the TCI + Morphine + SAHA and the TCI + SAHA group (*n* = 3 in each group). Analysis was based on mean gray values, normalized to β-actin. Data are expressed as the mean ± SEM. ^∗∗∗^*p* < 0.001 versus the Sham + Vehicle group, ^##^*p* < 0.01, ^###^*p* < 0001 versus the TCI + Vehicle group, and ^&&&^*p* < 0.001 versus the TCI + Morphine group.

Since HDACs can suppress gene transcription via promoting the deacetylation of histones, we further investigated whether inhibiting HDACs by SAHA could rescue the decreased MOR expression. When SAHA (20 mg/kg) was injected i.p. once daily from POD 8 to POD 14, both HDAC1 (**Figure 6A**) and HDAC2 (**Figure 6B**) expression in the DRG were significantly decreased. Conversely, the TCI-induced downregulation of MOR was significantly upregulated on POD 14 (**Figure 6C**). Furthermore, we examined the effects of SAHA on the expression of MOR, HDAC1, and HDAC2 in the DRG of TCI rats with morphine co-administration. When morphine (20 μg/kg) was injected, i.t. twice daily from POD 8 to POD 14, the Western blot analysis showed that HDAC1 (**Figure 6D**), HDAC2 (**Figure 6E**), and MOR (**Figure 6F**) expression in the DRG all maintained unchanged on POD 14 compared with TCI + Vehicle group. However, when morphine and SAHA were injected from POD 8 to POD 14, our data showed that both HDAC1 (**Figure 6D**) and HDAC2 (**Figure 6E**) expression was significantly decreased. Conversely, the MOR expression (**Figure 6F**) was significantly increased on POD 14.

### The Co-localization of HDACs and MOR in the DRG of the Sham + Vehicle, the TCI + Vehicle and the TCI + SAHA Rats

**FIGURE 7:**
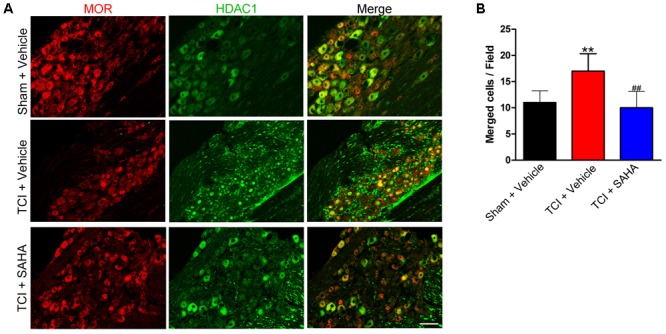
The co-localization of HDAC1 and MOR in the DRG of the Sham + Vehicle, the TCI + Vehicle and the TCI + SAHA group. **(A)** Double immunofluorescence staining images showing the co-localization of MOR (red fluorescence) and HDAC1 (green fluorescence) in the DRG of the Sham + Vehicle, the TCI + Vehicle and the TCI + SAHA group on POD 14. Scale bar: 100 μm. **(B)** Quantitative analysis of the number of double-labeled neurons in the DRG in all three tested groups (*n* = 5 in each group). Data are expressed as the mean ± SEM. ^∗∗^*p* < 0.01 versus the Sham + Vehicle group, ^##^*p* < 0.01 versus the TCI + Vehicle group.

Immunofluorescent staining showed that the distributions of MOR- and HDAC1-like immunoreactivities in the DRG in all three tested groups (**Figure 7A**). Consistent with the Western blot results, the immunofluorescence intensity of HDAC1 in the DRG was increased, while the immunofluorescence intensity of MOR was decreased on POD 14. Meanwhile, the immunofluorescent staining showed that HDAC1-like immunoreactivities were mainly located in the cytoplasm of the neurons in the DRG of Sham + Vehicle and TCI + SAHA group, while located in the nuclei of neurons in the TCI + Vehicle group (**Figure 7A**). Moreover, the number of double-labeled neurons was increased in the DRG of the TCI + Vehicle group on POD 14. When SAHA (20 mg/kg) was injected i.p. from POD 8 to POD 14, both MOR- and HDAC1-like immunoreactivities in the DRG recovered (**Figure 7A**). The quantitative analysis also showed that the number of double-labeled neurons in the DRG of the TCI + Vehicle group was significantly higher than that in the DRG of the Sham + Vehicle group. However, i.p. administration of SAHA reversed the increase in double-labeled neurons in the DRG (**Figure 7B**).

**FIGURE 8:**
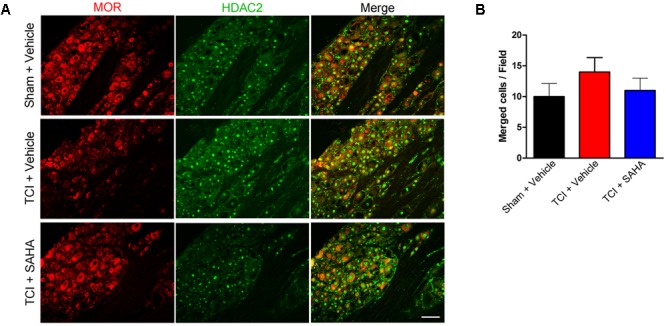
The co-localization of HDAC2 and MOR in the DRG of the Sham + Vehicle, the TCI + Vehicle and the TCI + SAHA group. **(A)** Double-staining immunofluorescence images showing the co-localization of MOR (red fluorescence) and HDAC2 (green fluorescence) in the DRG of the Sham + Vehicle, the TCI + Vehicle and the TCI + SAHA group on POD 14. Scale bar: 100 μm. **(B)** Quantitative analysis of the double-labeled neurons in the DRG in all three tested groups (*n* = 5 in each group). Data are expressed as the mean ± SEM.

Similar to the HDAC1 results, immunofluorescent staining showed the distribution of MOR- and HDAC2-like immunoreactivities in the DRG of all three tested groups (**Figure 8A**). Although the immunofluorescence intensity of MOR was decreased following TCI, the number of double-labeled neurons was unchanged in all three tested groups (**Figure 8A**). Consistently, the quantitative analysis also showed no significant difference in the number of double-labeled neurons in all three tested groups (**Figure 8B**).

## Discussion

Although the mechanism of morphine tolerance has been explored in several studies, most of these researches have explored the underlying mechanism based on normal conditions (Mayer et al., 1999; Hu et al., 2016; Sahbaie et al., 2016). However, the development of morphine tolerance under physiological conditions might be quite different from that under pathological conditions. It has been reported that the analgesic effects of morphine on BCP are much poorer than those on non-cancer pain, but the underlying mechanism is still unknown. In the current study, we showed that the weakened antinociceptive effects of morphine and rapid development of morphine tolerance during the pathogenesis of BCP might be correlated with the increased HDAC expression in the DRG. Our major findings were as follows: First, the increased HDAC expression was correlated with the decreased MOR expression in the DRG of TCI rats; and second, morphine tolerance occurred much earlier in TCI rats than in Sham rats. Co-administration of SAHA with morphine significantly delayed the occurrence of morphine tolerance in BCP. Third, SAHA administration reversed the upregulation of HDACs and restored TCI-induced decreased MOR expression in the DRG.

The analgesic effects of morphine mainly depend on MOR expression. Decreased MOR expression can contribute to the impaired analgesic effects of morphine and MOR agonists (Rashid et al., 2004; Kohno et al., 2005; Xu et al., 2014). In contrast, the upregulation of MOR by transgene expression in DRG neurons potentiates the antinociceptive effects of morphine (Xu et al., 2003; Zhang et al., 2016). Prior studies have demonstrated that MOR is downregulated in distinct populations of DRG neurons in BCP (Mao et al., 1995; Mercadante and Fulfaro, 2007; Yamamoto et al., 2008; Yao et al., 2016), whereas its expression is unchanged in the DRG under conditions of inflammatory pain (Kim et al., 2006; Yamamoto et al., 2008). This may explain why BCP is more resistant to morphine treatment than inflammatory pain (Luger et al., 2002; Zöllner et al., 2008; Zhao et al., 2015). In the present study, we found that MOR expression in the DRG was significantly decreased following TCI. When morphine was i.t. injected, the PWT of the TCI + Morphine group was increased at the beginning but subsequently decreased sharply. To confirm whether decreased MOR expression contributed to morphine tolerance in BCP, we then compared morphine tolerance in TCI rats and Sham rats. When morphine was i.t. injected twice daily from POD 8 to POD 14, the decreasing trend in the MPE line chart for TCI rats was significantly lower than that for Sham rats on POD 8 and POD 9. Considering that MOR expression was significantly lower in TCI rats than in Sham rats from POD 7, our data implied that morphine tolerance occurred more easily under pathological conditions of BCP than under physiological conditions, which might be due to the decreased MOR expression in the DRG of BCP.

Epigenetic changes are chemical modifications to chromatin that modulate gene activity without altering the DNA sequence (Wei, 2008). HDACs can remove the acetyl group from histones, which tightens chromatin structure and leads to decreased gene transcription (Wei, 2008). Accumulating evidence suggests that opioids and nociceptive signaling can converge on epigenetic mechanisms to enhance or prolong neural plasticity, which contributes to morphine tolerance and addiction (Wang et al., 2007; Sun et al., 2013; Sahbaie et al., 2016). Formisano et al. (2007) reported that ischemia could promote the deacetylation of core histone proteins H3 and H4 to silence MOR expression in hippocampal neurons. Our previous study also indicated that hypoacetylation of histone H3 and H4 promoted neuron-restrictive silence factor (NRSF) binding to the neuron-restrictive silencer element within the promoter area of the MOR gene, which drove the NRSF-induced downregulation of MOR transcription in the DRG (Zhu C. et al., 2017). In the current study, we found that the expression of HDAC1 and HDAC2 was negatively correlated with the expression of MOR in the DRG following TCI. The increased co-localization of HDAC1 and MOR was observed in TCI rats, which implied a close relationship between HDAC and MOR expression. Meanwhile, we found that HDAC1 was mainly located in the cytoplasm of neurons in the DRG of the Sham + Vehicle group and the TCI + SAHA group, while located in the nuclei of neurons in the DRG of the TCI + Vehicle group. However, it should be noted that the redistribution of HDAC1 from cytoplasm to nuclei still needs more evidences, such as immunofluorescent double staining of HDAC1 and DAPI or Western blot analysis of HDAC1 protein in nuclei and cytoplasm before and after TCI. The locations of different classes of HDACs are quite different from each other (Zhu Y. et al., 2017). Class II HDACs and HDAC1 are known to shuttle between the cytoplasm and the nuclei in physical and pathological conditions (Zhu Y. et al., 2017). Previous researches have indicated that HDAC1 is mainly located in neuronal nuclei in brain regions, such as hippocampus, to repress gene translation (Tao et al., 2017), but cytosolic HDAC1 could be observed in damaged axons in animal models of demyelination (Kim et al., 2010; Takase et al., 2013). Therefore, we hypothesized that the redistribution of HDAC1 might be associated with the pathogenesis of BCP. Our previous research has shown that HDAC1 could transport from cytoplasm to nuclei in the SDH of TCI rats (Hu et al., 2017). Therefore, the cytoplasm-nuclei translocation of HDAC1 in the DRG was consistent with our previous research. However, the underlying mechanism still needs further research. Furthermore, our data showed that SAHA administration reversed not only the upregulation of HDAC1 and HDAC2 but also TCI-induced downregulation of MOR in the DRG of TCI rats treated morphine or not. Collectively, these results indicate that morphine treatment could not influence the expression levels of HDACs in the DRG, and that the inhibition of HDACs could restore MOR expression levels in the DRG of TCI rats.

The effects of HDACi on morphine tolerance are controversial. Some studies have indicated that HDACi can enhance the analgesic effects of morphine and attenuate morphine tolerance (Dobashi et al., 2010; Tsai et al., 2016; Hou et al., 2017; Liao et al., 2018). However, some studies have demonstrated that chronic morphine treatment with simultaneous SAHA treatment can enhance opioid-induced hyperalgesia (Liang et al., 2013). In the present study, we found that systemic administration of SAHA (i.p., 20 mg/kg) combined with morphine (i.t., 20 μg/kg) produced a synergistically antinociceptive effect on BCP compared with SAHA or morphine alone. When morphine or SAHA was injected from POD 8 to POD 14, the pharmacological effects of morphine peaked immediately after the first dose, but its efficacy was decreased subsequently, whereas the analgesic effects of SAHA on BCP occurred following a pre-dosing treatment of 4 days. Thus, it appeared that the increased PWT of TCI + Morphine + SAHA rats from POD 11 to POD 14 compared with that of TCI + Morphine + Vehicle was due to the analgesic effects of pre-dosing SAHA. To rule out this possibility, we then analyzed the MPE which is one of the most widespread and convincing ways to evaluate morphine tolerance (Marcus et al., 2015; Corder et al., 2017). Our data showed that concomitant use of SAHA and morphine could improve the MPEs of TCI + Morphine rats, which suggested that morphine tolerance was attenuated by SAHA administration. Altogether, these results indicated that SAHA administration attenuated morphine tolerance in BCP. The different effects of SAHA on opioid-induced pain behavior might be due to different pathological conditions. Liang et al. studied the effects of SAHA on morphine-induced hyperalgesia in normal conditions (Liang et al., 2013), while our study and those by other researchers examined the effects of SAHA on morphine tolerance in the pathogenesis of chronic pain (Hou et al., 2017; Liao et al., 2018). HDAC expression and histone acetylation in pathological conditions may be quite different from those in physiological conditions. We believe that exploring the mechanism of morphine tolerance in pain states will provide more appropriate data for clinical applications. However, it should be noted that SAHA administration could not completely eliminate morphine tolerance; no significant difference was observed between the PWT of the TCI + Morphine + SAHA group and the TCI + SAHA + Vehicle group from POD 12 to POD 14. The reason might be lie in that morphine tolerance in BCP relies not only on MOR changes in the DRG but also on other mechanisms, such as neuroplasticity changes (Lutz et al., 2015; Trang et al., 2015).

Although many studies have interrogated the effects of SAHA on neurochemical changes in the DRG of BCP models via i.p. injection (Chiechio et al., 2009; Zammataro et al., 2014), the effects of SAHA on supra-spinal central nervous system (CNS) and SDH could not be ruled out. Previous research has indicated that i.p. SAHA injection could epigenetically increase *gad65* activities in the rat brain stem nucleus raphe magnus (Zhang et al., 2011). Our previous research also indicated that i.p. SAHA administration could down-regulate the expression levels of HDACs in the spinal dorsal horn (SDH) of TCI rats (data under submission). Thus, i.p. administration of SAHA can influence the supra-spinal CNS and SDH, which may confound our findings. Intrathecal or intraganglionic administration of SAHA may be a better way to circumvent these drawbacks. In our future study, we will study the underlying mechanism of attenuating morphine tolerance via intrathecal administration of SAHA.

The anti-tumor effects of SAHA on BCP may also obscure our findings. However, the anti-tumor effects of SAHA on BCP have been studied in our previous research. When SAHA (50 mg/kg) was injected i.p. from POD 1 to POD 14, the infiltration of cancer cells and severe osteolytic lesions were still easily observed in tibial marrow cavity, which suggested that SAHA administration could not inhibit cancer growth or cancer-induced bone destruction (data under submission). In the present study, 20 mg/kg SAHA was injected from POD 8 to POD 14. Either the dose or administration time of SAHA was less than that used in our previous research. Thus, we think the effects of SAHA on the analgesic effects of morphine could not be due to its anti-tumor effects.

Our previous research has indicated that the expression of NRSF, also called repressor element silencing transcription factor, was negatively correlated with MOR expression in a sarcoma murine model (Zhu C. et al., 2017). We found that NRSF could repress MOR gene translation via binding to the neuron-restrictive silencer element within the promoter area of the MOR gene. Furthermore, the NRSF activity was correlated with the hypoacetylation state of histone H3 and H4 (Zhu C. et al., 2017). Kim et al. (2004) also showed that NRSF-mediated MOR repression is sensitive to TSA, a HDAC inhibitor. Thus, we speculate that the increased HDACs may modulate NRSF activities to repress MOR expression in neurons in BCP models.

Repetitive use of morphine can easily cause analgesic tolerance, which hinders its prolonged use in clinical practice (King et al., 2005; Trang et al., 2015). Given that morphine is the mainstay for analgesic therapy in advanced cancer pain, there is an urgent need to explore the mechanisms underlying the easily occurred morphine tolerance in BCP and to find a way to attenuate such tolerance. The data in the current study suggest that HDACs may contribute to the impaired analgesic effects of morphine and easily occurred morphine tolerance by decreasing MOR expression in the DRG during the pathogenesis of BCP. And inhibition of HDACs by SAHA could improve the analgesic effects of morphine and attenuate morphine tolerance via restoring MOR expression in the DRG. These findings reveal a possible mechanism underlying morphine tolerance during the pathogenesis of BCP and provide a possible way to attenuate morphine tolerance in BCP.

## Author Contributions

All authors fulfill the authorship requirements and have approved the final version of the manuscript. Y-LD, Y-QL, and Z-XG: conceived and designed the experiments. X-TH, K-XZ, and W-JZ: performed the experiments. X-TH, CZ, and J-PD: analyzed the data. Y-LD, Y-QL, and F-MC: wrote or contributed to the writing of the manuscript.

## Conflict of Interest Statement

The authors declare that the research was conducted in the absence of any commercial or financial relationships that could be construed as a potential conflict of interest.
